# Correlation of Platelet Parameters With the Severity of Thrombocytopenia in Dengue Fever in Children Aged Less Than 18 Years at a Tertiary Care Centre: A Cross-Sectional Study

**DOI:** 10.7759/cureus.56829

**Published:** 2024-03-24

**Authors:** Rachana A Sontakke, Nisha R Aglave, Himanshu Dua

**Affiliations:** 1 Pediatrics, Narendra Kumar Prasadrao (NKP) Salve Institute of Medical Sciences and Research Center and Lata Mangeshkar Hospital, Nagpur, IND; 2 Pediatrics, Datta Meghe Medical College, Datta Meghe Institute of Higher Education and Research, Nagpur, IND

**Keywords:** platelet index, plateletcrit, platelet distribution width, mean platelet volume, thrombocytopenia, dengue fever

## Abstract

Introduction

Dengue is a tropical viral disease caused by dengue virus with varied severity ranging from dengue fever to dengue shock syndrome. In dengue infection, there is thrombocytopenia with platelet activation. According to various proposed theories, activation of platelets during thrombocytopenia leads to changes in platelet parameters like mean platelet volume (MPV), platelet distribution width (PDW), plateletcrit (PCT), and platelet index (PI). So, this study was conducted to correlate platelet parameters with the severity of thrombocytopenia in children with dengue fever at a tertiary care hospital.

Methods

An observational cross-sectional study was conducted on 72 children below 18 years admitted with dengue infection at a tertiary care hospital. All 72 patients were divided into three groups depending on platelet count. Group I included individuals with a platelet count less than 50000/mm^3^, while group II comprised patients with platelet counts ranging between 50001/mm^3^ and 100000/mm^3^, and group III encompassed individuals with platelet counts exceeding 100001/mm^3^. Platelet parameters like MPV, PDW, PCT, and PI were analyzed on day one and day three of admission. These parameters on day one and day three were correlated with the severity of thrombocytopenia in all three groups.

Results

PCT values on day one and day three were statistically significant (p<0.05) in all three groups with p-value <0.001 with profound positive correlation, which means PCT value decreases with increasing severity of thrombocytopenia. PI in group I was statistically significant on day one (p=0.009) but not on day three (p=0.063). PI in group II was statistically significant (p<0.05) on day three (p=0.002), while in group III, PI was significant statistically on day one (p<0.001). MPV in group I, on day one (p=0.006) and in group II, on day three (p= 0.049) were statistically significant (p<0.05). PDW was statistically significant only on day one (p=0.031) in group I, while was not significant in groups II and III.

Conclusion

MPV, PDW, and PCT increase with a decrease in platelet count, whereas there is an increase in PI. These platelet indices could be used to predict the severity of thrombocytopenia and severity of the dengue fever. Along with MPV and PDW, PCT could be used to assess the severity of the disease progression.

## Introduction

Because India is a tropical country, there is an epidemic of dengue cases yearly during monsoon season. The severity of illness in dengue is determined by the age of the child, infecting serotype, and any pre-existing disease. Thrombocytopenia is the most common laboratory finding. Thrombocytopenia in dengue is due to the suppression of bone marrow, platelet destruction by antibodies to the dengue virus, excessive consumption of platelets, viral replication-mediated destruction of platelets by complement-mediated lysis, and apoptosis [1]. In response to thrombocytopenia, bone marrow is activated and more giant cells are produced, which is reflected as an increase in mean platelet volume (MPV), which is a major response of bone marrow to thrombocytopenia [2].

In response to thrombocytopenia, bone marrow is activated and more giant cells are produced, which is reflected as an increase in MPV. MPV serves as a surrogate marker for bone marrow activity. Elevated MPV levels indicate a suitable response to thrombocytopenia, suggesting increased megakaryocyte activity. Conversely, reduced MPV levels are indicative of bone marrow suppression [3]. Platelet activation induces morphological alterations in platelets, leading to the formation of pseudopodia and associated structural modifications. These changes in platelet morphology can be observed in the platelet distribution width (PDW), which is a marker of volume variability as a result of platelet activation in thrombocytopenia [4]. Total platelet mass is indicated by measuring plateletcrit (PCT). A decrease in the platelet counts along with PCT and platelet index (PI) indicates excessive platelet consumption; an increase in these parameters suggests the recovery phase of dengue and the chances of complications related to thrombocytopenia will be less. PCT is the sum of platelet impulses, which are individually detected by impedance measurement [5]. The degree of thrombocytopenia predicts the severity of the disease, its complications, and prognosis in critically ill child [6]. This study is done to analyze the correlation of platelet parameters with the degree of thrombocytopenia in children with dengue fever. All the parameters can be easily calculated by routine cell counter reports without extra cost.

## Materials and methods

This was an observational cross-sectional study. The investigation is conducted on patients diagnosed with dengue fever at a tertiary care hospital within a rural setting. All children diagnosed with dengue infection between June 2022 and December 2022 and admitted to the hospital were enrolled in the study. Children below 18 years of age who were fulfilling diagnostic criteria as per WHO, along with nonstructural protein 1 (NS1) positive or serology marker immunoglobulin M (IgM), immunoglobulin G (IgG) positive were included in the study. The patients whose parents did not give consent; those with other diagnosed causes of fever; and those with preexisting hematological conditions like idiopathic thrombocytopenic purpura, platelet disorders, and malignancy were excluded from the study.

Prior to commencing the study, written informed consent was obtained from parents or guardians, and permission was secured from the institutional ethical committee at Narendra Kumar Prasadrao (NKP) Salve Institute of Medical Sciences and Research Center and Lata Mangeshkar Hospital with approval number of NKPSIMS & RC and LMH/IEC/13/2021. A comprehensive analysis of platelet parameters was conducted through a complete blood count (CBC) on both day one and day three of the patient's admission. This evaluation encompassed various platelet parameters like platelet count, MPV, PDW, PCT, and PI. Platelet count, MPV, and PDW values are directly available in the CBC report. PCT is calculated using the formula platelet count × MPV/10000 while PI is calculated using the formula MPV × PDW/Platelet count × PCT.

These parameters were meticulously calculated to gain insights into the patient's hematological profile. The collected data were subsequently categorized into three distinct groups based on platelet count ranges. Group I included individuals with a platelet count less than 50000/mm^3^, while group II comprised patients with platelet counts ranging between 50001 and 100000/mm^3^ and group III encompassed individuals with platelet counts exceeding 100001/mm^3^. This stratification allowed for a more nuanced understanding of the variations in platelet parameters among different cohorts of patients. By comparing the results between day one and day three within each group, clinicians could potentially discern trends, patterns, or abnormalities in platelet behavior over the course of the admission. The day one parameters were labeled as MPV-1, PDW-1, PCT-1, and PI-1, while the corresponding day three parameters were labelled as MPV-2, PDW-2, PCT-2, and PI-2. Such a detailed analysis aids in tailoring patient care strategies and addressing specific hematological challenges that may arise during the hospitalization period. Data was entered in Microsoft Office Excel 2019. To assess demographic differences, the student's t-test and ANOVA were employed for comparisons. Categorical data were subjected to a chi-square test to compare groups, while demographic data mean and standard deviation were determined. Correlation coefficient (r) was derived for each platelet parameter like MPV, PDW, PCT, and PI with severity of thrombocytopenia. 

## Results

During the study period, a total of 108 febrile patients underwent screening for dengue serology. Among them, 72 individuals who met the inclusion criteria were successfully enrolled in the study. Out of the 72 dengue patients, 27 (37%) fell within the six to nine years age group, 20 (28.8%) were aged between 16 and 18 years, while 10 (13.7%) and 15 (20.5%) were in the age groups of one to five and 10-15 years, respectively. Gender distribution revealed 37 (50.7%) females and 35 (49.3%) males, indicating no gender predilection (p=0.325).

In total 72 patients fulfilled the criteria and were enrolled in the present study. These patients were divided into three groups as per their platelet count. Group I consisted of patients with a platelet count of <50000/mm^3^, group II had a count of 50001 to 100000/mm^3^ while group III had a count of >100001/mm^3^. Out of 72 patients, 19 (26.38%), 22 (30.55%), and 31 (43.05%) were in group I, group II, and group III, respectively. Out of the total patients in group I, five (26.31%) progressed to dengue hemorrhagic fever (DHF) or dengue shock syndrome (DSS). From group II, three (13.63%) patients progressed to DHF/DSS whereas no patient progressed to DHF/DSS from group III (Table 1).

**Table 1:** Number of patients in each group progressing to DHF/DSS Group I: patients with platelet count <50000/mm^3^; group II: patients with platelet count <50001-100000/mm^3^; group III: patients with platelet count >100001/mm^3^. DHF, dengue hemorrhagic fever; DSS, dengue shock syndrome; n, total number of enrolled patients; %, percentage

Platelet count	Dengue fever	Progressed to DHF/DSS
Group I	19 (26.38%)	5 (26.31%)
Group II	22 (30.55%)	3 (13.63%)
Group III	31 (43.05%)	0 (0%)
Total (n)	72	8 (11.11%)

In group I, patients with platelet counts less than 50000/mm^3^, platelet indices like MPV, PDW, PCT, and PI were compared on day one and day three of admission. PCT on day one (p<0.001) and on day three (p<0.001) and PI on day one (p=0.009) were statistically significant (p<0.05). MPV on day one (p=0.006) and PDW on day one (p=0.031) were statistically significant (p<0.05), whereas MPV (p=0.703) and PDW (p=0.609) on day three were statistically not significant (Table 2).

**Table 2:** Comparison of platelet parameters with platelet counts <50000 (group I) P<0.05 is considered as statistically significant. Day one parameters: MPV-1, PDW-1, PCT-1, and PI-1; day three parameters: MPV-2, PDW-2, PCT-2, and PI-2. MPV, mean platelet volume; PDW, platelet distribution width; PCT, plateletcrit; PI, platelet index; SD, standard deviation

Platelet parameters	Mean	Standard deviation	Correlation coefficient	P-value
MPV-1	9.41	2.159	0.604	0.006
MPV-2	9.48	1.923	-0.094	0.703
PDW-1	65	27.1406	0.496	0.031
PDW-2	51.874	18.241	0.125	0.609
PCT-1	0.02455	0.015404	0.936	<0.001
PCT-2	0.10153	0.109192	0.98	<0.001
PI-1	0.11	0.14	-0.581	0.009
PI-2	0.04	0.06	-0.434	0.063

In group II, patients with platelet counts between 50001 and 100000/mm^3^, platelet indices like MPV, PDW, PCT, and PI were compared on day one and day three of admission. PCT on day one (p<0.001) and day three (p<0.001) showed statistical significance (p<0.05), while PI (p=0.002) and MPV (p=0.049) on day three were statistically significant (p<0.05). PDW on day one (p=0.557) and on day three (p=0.923) were not statistically significant (Table 3).

**Table 3:** Comparison of platelet parameters with platelet counts <50001-100000 (group II) P<0.05 is considered as statistically significant. Day one parameters: MPV-1, PDW-1, PCT-1, and PI-1; day three parameters: MPV-2, PDW-2, PCT-2, and PI-2. MPV, mean platelet volume; PDW, platelet distribution width; PCT, plateletcrit; PI, platelet index; SD, standard deviation

Platelet parameters	Mean	Standard deviation	Correlation coefficient	P-value
MPV-1	9.073	1.2491	-0.025	0.991
MPV-2	8.664	1.3117	-0.425	0.049
PDW-1	59.7	19.7626	-0.123	0.557
PDW-2	55.714	16.6830	0.022	0.923
PCT-1	0.06359	0.019063	0.692	<0.001
PCT-2	0.13977	0.094654	0.980	<0.001
PI-1	0.013	0.012	-0.623	0.093
PI-2	0.009	0.013	-0.366	0.002

In group III, patients with platelet counts more than 100001/mm^3^, platelet indices like MPV, PDW, PCT, and PI were compared on day one and day three of admission. PCT on day one (p<0.001) and day three (p<0.001) showed statistical significance (p<0.05), while PI on day one (p<0.001) was statistically significant (p<0.05). MPV on day one (p=0.159) and MPV on day three (p=0.835), PDW on day one (p=0.809) and PDW on day three (p=0.609) were not statistically significant (Table 4).

**Table 4:** Comparison of platelet parameters with platelet counts >100001 (group III) P<0.05 is considered as statistically significant. Day one parameters: MPV-1, PDW-1, PCT-1, and PI-1; day three parameters: MPV-2, PDW-2, PCT-2, and PI-2. MPV, mean platelet volume; PDW, platelet distribution width; PCT, plateletcrit; PI, platelet index; SD, standard deviation

Platelet parameters	Mean	Standard deviation	Correlation coefficient	P-value
MPV-1	8.451	1.0365	-0.255	0.159
MPV-2	9.291	1.5304	0.038	0.835
PDW-1	58.775	15.2545	-0.045	0.809
PDW-2	54.784	21.9497	-0.094	0.609
PCT-1	0.11503	0.077080	0.959	<0.001
PCT-2	0.17091	0.087842	0.963	<0.001
PI-1	0.002	0.0015	0.632	<0.001
PI-2	0.030	0.108	-0.283	0.117

## Discussion

Dengue fever is one of the major health problems in central India. WHO includes thrombocytopenia as a pointer for the clinical severity of the disease. Various platelet indices are available, which suggest the change in shape, size, and volume of platelets. These indices can be used to predict the severity of thrombocytopenia. This will help in aggressive monitoring and timely intervention to avoid complications and reduce mortality. In this study, a total of 72 patients were included, among whom the majority, comprising 27 (37%), fell within the age range of six to nine years. Following closely, 20 (28.8%) of the participants belonged to the age group of 16-18 years. The study, conducted in Delhi, mirrored these findings, demonstrating a comparable incidence of dengue fever, 44.11% among children aged five to 10 years [7]. There were 37 (50.7%) females and 35 (49.3%) males, showing no gender predilection, which was statistically not significant. In contrast, other studies indicated male preponderance [8]. These 72 patients were categorized into three groups: group I with platelet counts <50000 mm^3^, group II with platelet counts 50001 to 100000/mm^3^, and group III with platelet counts >100001/mm^3^. Among groups I and II, five (26.31%) and three (13.63%) of patients, respectively, progressed to DHF/DSS, while none from group III developed DHF/DSS. These results align with findings from another study, which demonstrated an increased incidence of DHF/DSS in patients with decreased platelet count [9].

The normal value of MPV is between 8.6 and 15.5 fL. In this study, the mean MPV on day one and day three of admission in all three groups was in the normal range. MPV-1 in group I was increased compared to that in groups II and III and it was statistically significant, showing moderate positive correlation. Mean MPV-2 did not show any significant change with increasing platelets in all three groups. In a similar study, the MPV fell within the normal range across all three groups (I, II, and III). The mean values were 10.29±1.56, 10.90±2.45, and 10.79±3.35, respectively [10]. In a study by Kiran et al., mean MPV values were 9.2 fL, 12 fL, and 13.8 fL in patients with platelet count groups below 20000, 20000-100000, and more than 100000, respectively [11]. Likewise, Dewil et al. observed a direct proportionality between MPV and platelet count in dengue fever. A robust correlation was found between low MPV (<9 fL) and low platelet count, mirroring our study's findings [12]. We noticed a correlation coefficient (r) of 0.124 and a p-value of <0.05 [1]. The normal value of PDW is between 8.3 fL and 56.6 fL. Mean PDW-1 was increased than normal in all three groups. Mean PDW-1 in groups I, II, and III were 65, 59.7, and 58.775, respectively. Mean PDW-1 was highest in group I, which was statistically significant and showed a mild positive correlation. Mean PDW in all three groups was reduced with increasing platelet count but was not statistically significant. Nehara et al. proposed that an elevated PDW is more sensitive for detecting DHF and exhibits higher specificity for dengue fever [13]. Similar to the study by Jacob et al., this study found that PDW exhibited a negative correlation with a decrease in platelet count, although the correlation coefficient was not statistically significant (-0.038). PDW showed a trend similar to platelet count and an inverse relationship to MPV [4]. 

The normal value of PCT is between 0.22% and 0.24%. The mean value of PCT-1 was reduced in all three groups but was greatly reduced in group I with severe thrombocytopenia. These trends were statistically significant along with a profound positive correlation. The mean value of PCT-2 in each group was increased with the improvement in platelet counts. This also showed a profound positive correlation and was statistically significant [13]. The PCT was consistently low across all three groups, with the lowest value observed in group III, having a mean value (0.06±0.05) [10]. There was a positive correlation observed between PCT and low platelet count, with a correlation coefficient (r) value of 0.238 and a p-value of 0.05. In a different investigation by Hardeva et al., it was discovered that patients with dengue fever had platelet counts that were just below normal values [14]. The mean PCT was 0.11 (0.031%) among deceased patients and 0.17 (0.048%) in discharged patients, showing a significant difference between the mean values of PCT (p=0.017). A PCT value of <0.11% had 91% sensitivity and a 95% specificity in mortality prediction [15].

In this study, PI-1 was raised in group I with severe thrombocytopenia, which was statistically significant and showed a moderate negative correlation. PI-2 showed a decreasing trend with an increase in platelet count. In this study, group I, a decrease in platelet count and PCT is associated with an increase in PI. Decreasing PCT is seen in group I, which was a group with severe thrombocytopenia and severe dengue ​[11]. MPV and PWD are being used for the analysis of platelet trends in critical patients. Our study showed that over MPV and PDW, PCT showed a profound positive correlation with platelet count in all three groups. A decrease in platelet counts and decrease in PCT are associated with increase in PI.

Simple and readily available CBC analysis will provide the required information like platelet counts, MPV, and PDW, while other indices like PCT and PI are derived from this. Bedside calculation of PCT and PI would help us to find out the trends of platelets and help for early recognition of those patients with severe disease so that aggressive monitoring and timely management will reduce mortality. This study has certain limitations like the sample size is less. The strength of the study could have been improved by increasing the sample size. Also, in this study, there was no mortality but other clinical parameters like length of hospital stay, the need for admission to the intensive care unit, and the need of platelet transfusion were not taken into consideration.

## Conclusions

In this study, thrombocytopenia was found to be associated with an increase in MPV and PDW parameters. Additionally, thrombocytopenia correlated with a decrease in PCT, while there was an increase in PI. As the illness progressed and platelet count improved, these platelet parameters normalized. When compared to other parameters, PCT and PI exhibited strong statistical significance with thrombocytopenia. These platelet parameters and indices can be used to predict the severity of thrombocytopenia and its complications. Implementing the use of such parameters could help segregate those patients who need aggressive monitoring, and timely management can be initiated to reduce mortality.
